# Supplementary material for: Multiple Immunostainings with Different Epitope Retrievals—The FOLGAS Protocol
Source: Int J Mol Sci. 2021 Dec 25;23(1):223. doi: 10.3390/ijms23010223 (PMC8745613; doi:10.3390/ijms23010223)
Supplement: Supplementary file 1 [file ijms-23-00223-s001.zip › Figures.pdf]

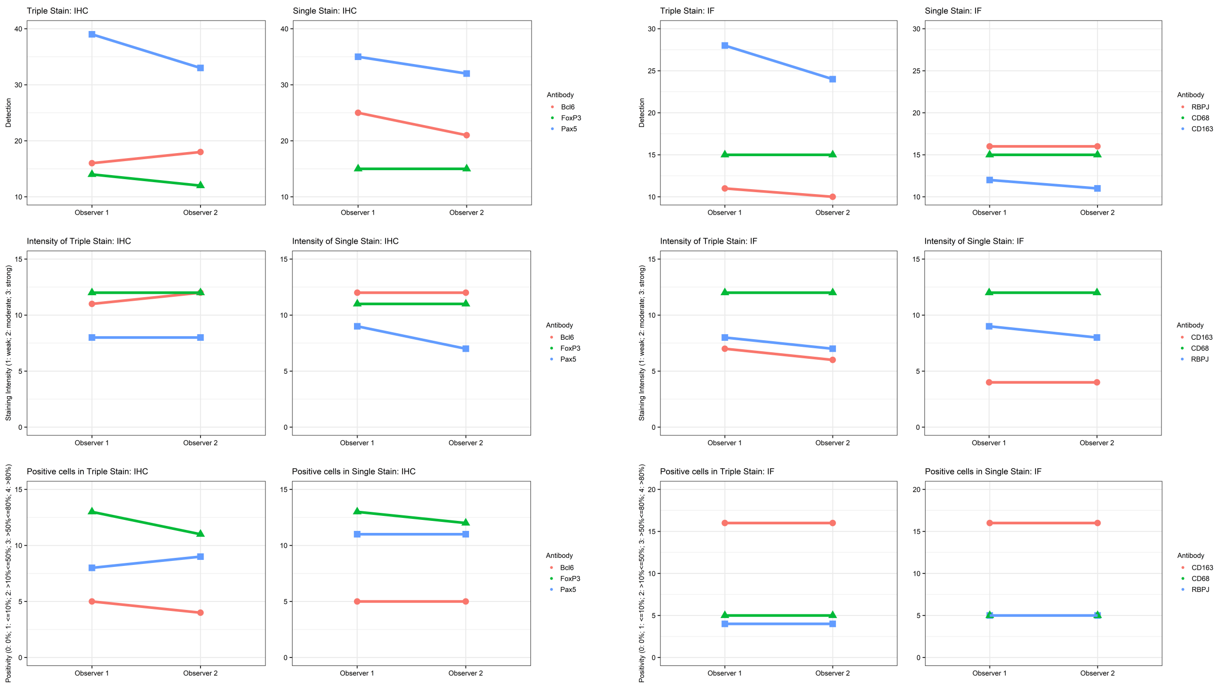

Figure S1. Overview of sums of H-scores, sums of intensity and sums of positive cells, semiquantitatively evaluated by two independent observers.

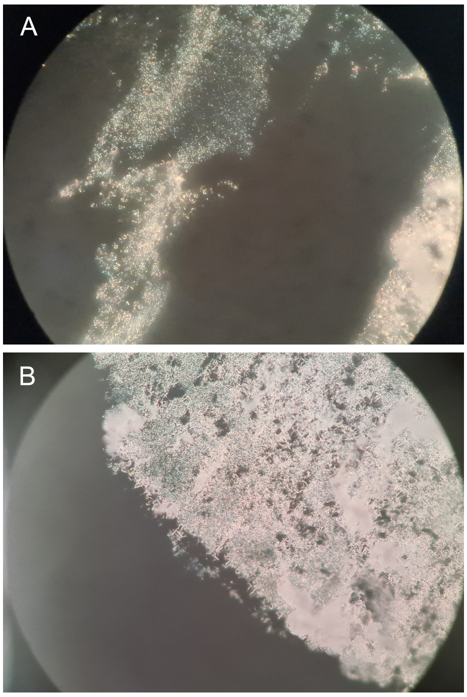

Figure S2. Birefringent paraffin precipitations. A) Xylene was saturated with paraffin, a droplet of this solution was pipetted on an objective plate, directly followed by rinsing with 100% ethanol, resulting in direct precipitation of small birefringent paraffin “crystals”. Further rinsing with ethanol does not dilute the precipitations. B) Rinsing with acetone does not dilute the precipitations. However, larger fragments could be mobilized from the glass objective plate. 20x original magnification for A and B.
