# Supplementary material for: Multiple Immunostainings with Different Epitope Retrievals—The FOLGAS Protocol
Source: Int J Mol Sci. 2021 Dec 25;23(1):223. doi: 10.3390/ijms23010223 (PMC8745613; doi:10.3390/ijms23010223)
Supplement: Supplementary file 1 [file ijms-23-00223-s001.zip › TableS1.pdf]

Observer 1 (SK)

Immunohistochemistry												
	Germinal Center			Mantle Zone			Interfollicular Area			Epithelium		
Single Staining	Positive Cells	Staining Intensity	Score	Positive Cells	Staining Intensity	Score	Positive Cells	Staining Intensity	Score	Positive Cells	Staining Intensity	Score
Bcl6	3	3	9	2	2	4	2	2	4	4	2	8
FoxP3	1	3	3	1	3	3	2	3	6	1	3	3
PAX5	4	2	8	4	3	12	2	3	6	3	3	9
Triple Staining												
Bcl6	3	3	9	1	2	2	1	2	2	3	1	3
FoxP3	1	2	2	1	3	3	2	3	6	1	3	3
PAX5	4	3	12	4	3	12	2	3	6	3	3	9

Immunofluorescence				
	Germinal Center	Mantle Zone	Interfollicular Area	Epithelium

Single Staining	Positive Cells	Staining Intensity	Score	Positive Cells	Staining Intensity	Score	Positive Cells	Staining Intensity	Score	Positive Cells	Staining Intensity	Score
CD163 (555)	1	2	2	1	1	1	2	3	6	1	3	3
CD68 (Aqua)	1	3	3	1	3	3	2	3	6	1	3	3
RBPJ (488)	4	1	4	4	1	4	4	1	4	4	1	4
Triple Staining												
CD163 (555)	1	2	2	0	0	0	2	3	6	1	3	3
CD68 (Aqua)	1	3	3	1	3	3	2	3	6	1	3	3
RBPJ (488)	4	1	4	4	2	8	4	2	8	4	2	8

Observer 2 (MS)

Immunohistochemistry												
	Germinal Center			Mantle Zone			Interfollicular Area			Epithelium		
Single Staining	Positive Cells	Staining Intensity	Score	Positive Cells	Staining Intensity	Score	Positive Cells	Staining Intensity	Score	Positive Cells	Staining Intensity	Score
Bcl6	3	3	9	2	1	2	2	1	2	4	2	8
FoxP3	1	3	3	1	3	3	2	3	6	1	3	3
PAX5	4	2	8	4	3	12	2	3	6	2	3	6
Triple Staining												
Bcl6	3	2	6	1	2	2	1	2	2	4	2	8
FoxP3	1	3	3	1	3	3	1	3	3	1	3	3
PAX5	3	3	9	4	3	12	2	3	6	2	3	6

Immunofluorescence				
	Germinal Center	Mantle Zone	Interfollicular Area	Epithelium

Single Staining	Positive Cells	Staining Intensity	Score	Positive Cells	Staining Intensity	Score	Positive Cells	Staining Intensity	Score	Positive Cells	Staining Intensity	Score
CD163 (555)	1	1	1	1	1	1	2	3	6	1	3	3
CD68 (Aqua)	1	3	3	1	3	3	2	3	6	1	3	3
RBP1 (488)	4	1	4	4	1	4	4	1	4	4	1	4
Triple Staining												
CD163 (555)	1	1	1	0	0	0	2	3	6	1	3	3
CD68 (Aqua)	1	3	3	1	3	3	2	3	6	1	3	3
RBP1 (488)	4	1	4	4	2	8	4	1	4	4	2	8

Suppl. Table S1. Evaluations of positive cells and staining intensities for single stainings and corresponding triple stainings, respectively. Semiquantitative evaluations were as follows: Relative amount of positive cells: 0: 0%; 1: ≤10%; 2: >10%≤50%; 3: >50%≤80%; 4: >80%. Staining intensity: 1: weak; 2: moderate; 3: strong. Score was calculated by multiplying positive cells with staining intensity.
